# Clinical Experience of Ceftaroline Fosamil in Gram-Positive Infective Endocarditis: A Multicenter Real-World Observational Study

**DOI:** 10.3390/antibiotics15050466

**Published:** 2026-05-05

**Authors:** Daniel Arnés-García, Jorge Calderón-Parra, Marina Calvo-Salvador, Carmen Herrero-Rodríguez, Svetlana Sadyrbaeva-Dolgova, Carmen Hidalgo-Tenorio

**Affiliations:** 1 Internal Medicine Department, Hospital Universitario Virgen de las Nieves, 18014 Granada, Spain; 2 Infectious Diseases Unit, Hospital Puerta de Hierro de Majadahonda, 28222 Madrid, Spain; jparra@salud.madrid.org; 3 Pharmacy Department, Hospital Puerta de Hierro de Majadahonda, 28222 Madrid, Spain; mcalvos@salud.madrid.org; 4 Infectious Diseases and Microbiology Unit, Complejo Hospitalario de Jaén, 23007 Jaén, Spain; carmen.herrero.sspa@juntadeandalucia.es; 5 Pharmacy Department, Hospital Universitario Virgen de las Nieves, Instituto de Investigación Biosanitario de Granada (IBS-Granada), 18014 Granada, Spain; svetlana.sadyrbaeba.sspa@juntadeandalucia.es; 6 Infectious Diseases Unit, Hospital Universitario Virgen de las Nieves, Instituto de Investigación Biosanitario de Granada (IBS-Granada), 18014 Granada, Spain

**Keywords:** ceftaroline fosamil, *Staphylococcus aureus*, infective endocarditis, real-world study

## Abstract

**Background/Objectives:** Ceftaroline fosamil (CFT) is a fifth-generation cephalosporin approved in Spain for skin and soft tissue infections and community-acquired pneumonia. CFT may also be useful against endovascular infections. This real-world study aimed to evaluate its effectiveness and safety in patients with Gram-positive (GP) infective endocarditis (IE). **Methods:** This observational, retrospective multicenter study enrolled adults with GP-IE treated with CFT for ≥48 h. Recruitment extended from CFT incorporation in participating hospitals through May 2024. Data were gathered on demographic, clinical, and microbiological variables, adverse effects, overall and IE-related mortality, relapses, and a composite unfavorable outcome. **Results:** Seventy-six patients (65.8% male) were enrolled, with a mean age of 68.9 ± 12.8 years and an age-adjusted Charlson index of 4; 55.3% had previous moderate/severe valvular heart disease, 35.5% had atrial fibrillation, 34.2% chronic heart failure, 17.1% chronic kidney disease, and 22.4% septic shock. IE was native valve-related in 53.9%, involving the aortic valve in 38.2% and the mitral in 30.3%. *Staphylococcus aureus* was isolated in 48.7%, being methicillin-resistant in 40.5% of cases. CFT was salvage therapy in 65.8% and combined with other antibiotics in 94.7%. Valve replacement was indicated in 64.5% but performed in only 67.3% of these. At six months, the adverse effect rate was 9.2%, overall crude mortality 38.2%, infection-related mortality 28.9%, and composite unfavorable outcome 40.1%. In multivariate analysis, mortality-related factors were age-adjusted Charlson index, septic shock, and methicillin-sensitive *S. aureus*. **Conclusions:** CFT showed favorable outcomes and acceptable safety in the real-life treatment of GP-related IE in clinically complex patients with high comorbidity and previous antibiotic therapy failures.

## 1. Introduction

The incidence of infective endocarditis (IE) has increased worldwide over the past decade, reaching an annual rate of more than seven cases per 100,000 persons in Spain [1]. Around one in five patients with IE require surgery for cardiac, musculoskeletal, neurologic, renal, and/or systemic infectious complications, including septic emboli and mycotic aneurysms [1]. Despite diagnostic and therapeutic advances, IE has a high in-hospital mortality rate of almost 20% in Spain [1], where the percentage of recurrence ranges from 5 to 10% [2].

IE is mainly caused by Gram-positive (GP) microorganisms, notably *Streptococci*, *Staphylococci* and, to a lesser extent, *Enterococci* [3]. The therapeutic arsenal against this disease has expanded over recent years to include novel antibiotics with activity against GP microorganisms, such as the fifth-generation cephalosporins ceftaroline fosamil (CFT) and ceftobiprole medocaril and the long-acting antibiotics dalbavancin and oritavancin [4].

CFT exerts its bactericidal action by inhibiting peptidoglycan penicillin-binding protein 2a through high-affinity binding [5]. Like other cephalosporins, its activity depends on the length of time it remains above the minimum inhibitory concentration [6]. Its spectrum against GP includes *Streptococcus pneumoniae* (including those resistant to third-generation cephalosporins); coagulase-negative *Staphylococci*; and *Staphylococcus aureus*, either sensitive (MSSA) or resistant (MRSA) to methicillin [7]. Its spectrum against Gram-negative bacteria is comparable to that of third-generation cephalosporins [8]. The European Medicines Agency has approved its use for community-acquired pneumonia and for skin and soft tissue infections [9,10]. Its pharmacokinetics and pharmacodynamics may make CFT potentially useful against bacteremia, IE, nosocomial pneumonia, or osteoarticular infections [11,12], especially in cases of therapeutic failure or resistance to other antibiotics such as daptomycin or linezolid [13].

Although no randomized clinical trials have yet been reported on the comparative efficacy of CFT against IE, various observational studies have been published [14]. In the USA, a one-month survival rate over 70% was reported in a retrospective study of the CAPTURE cohort of 55 CFT-treated patients with IE, despite its administration as salvage therapy in more than 90% of cases [15]. A more recent US study of 70 patients recorded a similar level of clinical success, although only patients with IE due to MRSA were enrolled, and the study population included a high percentage with right-sided involvement and a large proportion of injection drug users [16]. Comparable outcomes were obtained in a retrospective Spanish study of 70 CFT-treated patients with IE [17]. However, the single-center design and specific patient populations of these studies limit the generalizability of their findings, and multi-center investigations are needed to fully capture the clinical heterogeneity of patients with IE.

With this background, the present multi-center study was designed to evaluate the effectiveness and safety of CFT administered to a wide range of patients with IE in a real-world clinical setting. A second objective was to assess the influence on six-month mortality rate of the characteristics of the patients and the modality of CFT administration (e.g., monotherapy or combination therapy, first-line or salvage), among other clinical factors.

## 2. Results

### 2.1. Cohort Description

The study cohort comprised 76 patients (65.8% male) with a mean age of 68.9 ± 12.8 years and an age-adjusted Charlson index of 4 (P25–P75: 3–6). Among patients, 55.3% had previous moderate or severe valvular heart disease, 35.5% had atrial fibrillation, 34.2% chronic heart failure, 17.1% chronic kidney disease, 13.2% chronic obstructive pulmonary disease, and 9.2% drug-induced immunosuppression. Results for the remaining demographic and clinical variables at baseline are summarized in Table 1.

Definite IE was diagnosed in 88.2% of the patients. At diagnosis, 22.4% of patients were in septic shock requiring vasopressor support, and 53.9% had septic emboli. Native valves were involved in 53.9% of cases, late prosthetic valves in 23.7%, and early prosthetic valves in 14.5%. The aortic valve was involved in 38.2% and the mitral valve in 30.3%, while multivalvular IE was recorded in 15.8%. Other IE characteristics are reported in Supplementary Table S1.

### 2.2. Microbiological Isolates

GP cocci accounted for 97.4% of microbiological isolates. The most frequently isolated organism was *S. aureus* (48.7%), and 40.5% of these (15/37) were methicillin-resistant. Coagulase-negative staphylococci were isolated in 42.1%, predominantly *S. epidermidis* (32.9%). Further microbiological details are exhibited in Supplementary Table S2.

### 2.3. Antimicrobial Regimen and Valve Replacement

CFT was administered as an empirical treatment in 34.2% of cases due to clinical suspicion of GP IE and as targeted treatment in the remaining 65.8% after identification of the microorganism. CFT was administered as salvage therapy in 65.8% of cases and first-line treatment in the remaining 34.2%.

The reasons for CFT administration as salvage therapy were previous antibiotic therapy failure in 42.1%, adverse effects in 11.8%, and modification after antibiotic sensitivity testing in 10.5%. Combined antibiotic regimens (66% daptomycin, 50% beta-lactams, 16% vancomycin) had been received by 50% of the patients administered with CFT as salvage therapy. The median duration of previous antibiotic therapy was 7 days (P25–P75: 4–9.25).

CFT was predominantly administered in combination with other antibiotics (94.7%). Among combined antibiotic regimens, the most frequently used was CFT and daptomycin dual therapy (87.9%) (Table 2). The median duration of CFT treatment was 14 days (P25–P75: 6–24), with a median cumulative dose of 17.5 g (P25–P75: 8.6–34.5).

In 36.8% of IE cases, CFT treatment was interrupted or switched to another antibiotic during hospitalization. This was due to clinical improvement that allowed treatment simplification in 15.8%, de-escalation based on microbiological findings in 10.5%, or CFT-related adverse effects in 7.9%. The entire course of antibiotic treatment for IE was completed with CFT in 11.9% of patients, while it was followed by oral or long-acting parenteral antibiotics in 28.9% of patients for consolidation-phase completion or as a chronic treatment.

Valve replacement was indicated in 64.5% of the patients but was not performed in 32.7%, largely due to a high comorbidity burden and poor functional status (56.3%) or hemodynamic instability (31.3%).

### 2.4. Health Outcomes

The median length of hospital stay was 33 days (P25–P75: 19.25–48.75). The total crude mortality rate was 38.2% at six months, while the cumulative infection-related mortality rate was 13.2% at 14 days, 23.7% at 28 days, and 28.9% at six months. The non-IE-related mortality rate was 9.2% (Table 3). The composite unfavorable outcome rate was 40.1%. Three patients (3.9%) were lost to follow-up and excluded from the outcome analyses. A single case of relapse was recorded in a patient with native valve IE due to MRSA at three months, manifesting as early prosthetic IE, despite valve replacement surgery and completion of antibiotic treatment.

### 2.5. Adverse Effects

Adverse effects potentially attributable to CFT were reported in seven patients (9.2%) and prompted discontinuation of the antibiotic in six of these (7.9%). The effects were severe in two patients (2.6%), moderate in four (5.2%), and mild in one (1.3%).

Reported adverse effects included neutropenia (2.6%); nausea, vomiting, or diarrhea (2.6%); urticaria (1.3%); interstitial nephritis (1.3%); and *Clostridioides difficile* infection (1.3%). Both cases of neutropenia were classified as severe (minimum neutrophil counts of 200 and 400 cells/µL) and resolved within one week after CFT discontinuation. Serum creatinine levels increased to 3.7 mg/dL in the case of interstitial nephritis but returned to baseline values (1.8 mg/dL) within eight days after CFT discontinuation and corticosteroid administration (Supplementary Table S3). The mild adverse effect in one patient was a self-limited episode of vomiting/diarrhea that did not require CFT dose adjustment or discontinuation.

### 2.6. IE-Related Mortality Risk Factors

In bivariate analyses, IE-related mortality was significantly associated with higher age-adjusted Charlson Comorbidity Index (6.1 vs. 3.7, *p* = 0.0001) and greater frequency of intensive care unit admission (54.5% vs. 15.7%, *p* = 0.001), sepsis/septic shock (50.0% vs. 23.5%, *p* = 0.025), persistent bacteremia (18.2% vs. 0%, *p* = 0.008), moderate/severe chronic kidney disease (36.4% vs. 7.8%, *p* = 0.003), ischemic heart disease (40.9% vs. 11.8%, *p* = 0.005), chronic lower limb ischemia (18.2% vs. 2.0%, *p* = 0.027), MSSA IE (45.5% vs. 21.6%, *p* = 0.039), and non-performance of indicated surgery (40.9% vs. 9.8%, *p* = 0.002) (Table 4). In the parsimonious multivariate logistic regression model, age-adjusted Charlson Comorbidity Index (OR 1.66; 95% CI 1.24–2.23), septic shock (OR 5.42; 95% CI 1.38–21.36), and MSSA IE (OR 3.85; 95% CI 1.11–13.39) remained independently associated with IE-related mortality (Table 4).

In a bivariate sub-analysis of patients with IE due to MSSA versus other GP microorganisms, the former had a higher frequency of sepsis and septic shock (50.0% vs. 22.2%, *p* = 0.017) and a higher non-performance rate of indicated surgery (36.4% vs. 14.8%, *p* = 0.037) (Supplementary Table S4).

## 3. Discussion

This real-world study has the largest multicenter cohort of CFT-treated patients with GP-IE published to date. It is characterized by advanced age, with a mean age above 65 years, and a high burden of comorbidities, particularly cardiovascular disease. More than half of the IE episodes involved native valves, predominantly left-sided, and *S. aureus* was the most frequently implicated microorganism. This profile is consistent with epidemiological trends observed in patients with IE over the past decade [18,19]. The clinical severity of the present cohort was elevated, given that septic emboli were observed in more than half of the patients and septic shock in around a quarter.

CFT was mainly used as a salvage therapy and in combination with other antibiotics after previous treatment failure or intolerance to conventional regimens, reflecting a complex clinical scenario. CFT was most frequently combined with daptomycin, based on demonstrations of the bactericidal synergy of this combination in in vitro [20], animal [21], and human [22,23] studies and the faster bacteremia clearance obtained.

The duration of treatment and the total cumulative dose of CFT varied among these patients. CFT remained part of the antibiotic regimen until completion in slightly more than one-tenth of patients, and it was discontinued after clinical improvement or antibiotic de-escalation in just over one-third. Valve replacement was indicated in 64.5% of patients, similar to the percentage reported in the European endocarditis registry (EURO-ENDO) [19]. However, surgery was ultimately performed in just over two-thirds of these cases (43.4% of the overall cohort), which is slightly lower than the rates reported in the EURO-ENDO and the International Collaboration on Endocarditis (ICE) cohorts, where surgical approach exceeded 50% [19,24].

CFT demonstrated a favorable tolerability profile. The rate of adverse events potentially attributable to the drug was 9.2%, and all were reversible after CFT discontinuation. This is comparable to the rate described by Brandariz-Núñez et al. [17] and slightly higher than the rate in larger cohorts of patients with different infectious syndromes, which was below 4% [25,26]. Between-study variations in adverse effect rates may be explained by differences in cumulative dosage, treatment duration and/or the antibiotics used in combined therapies.

The total mortality rate was 38.2% and the infection-related mortality was 28.9%. In both cases, the mortality was concentrated within the first 28 days. These elevated percentages contrast with the mortality of around 20% reported in large international IE registries (EURO-ENDO, ICE) [19,24]. However, account should be taken of the advanced age of the patients, the high comorbidity burden, the elevated rates of *Staphylococcus aureus* infection and septic shock in the cohort, and the substantial proportion of patients who did not receive surgery when indicated. These factors have all been associated with a worse prognosis in IE [27,28,29].

Outcomes were comparable to those in previous observational studies on CFT in IE, which published clinical success rates of around 70% [15,16,17]. The composite unfavorable outcome rate was 40.1%. This endpoint has not been considered in previous publications on IE treatment but was adopted in the present study as a more clinically relevant and comprehensive measure of the response to therapy. In this way, it considers not only survival but also relapses and CFT discontinuance due to treatment failure or toxicity.

The IE-related mortality of the patients was mainly influenced by the baseline comorbidity burden, the clinical severity of the episode, and the causative microorganism. In multivariate analysis, the Charlson index, presence of septic shock, and MSSA infection were independently associated with mortality.

In comparison to IE caused by other Gram-positive cocci, IE due to either MSSA or MRSA has consistently been associated with greater virulence, higher bacterial inoculum, an increased frequency of septic emboli, and a more severe clinical presentation [30,31,32]. The association between MSSA infection and higher mortality observed in our cohort is unexpected and should be interpreted with caution. In the present study, IE due to MSSA was associated with higher frequencies of sepsis and the non-performance of indicated surgery in comparison to IE caused by other GP microorganisms. It is possible that the contraindication of essential surgery due to clinical severity, suggesting the presence of indication bias, may be responsible for the excess mortality observed.

The study limitations include its retrospective observational design with no comparator group, which precludes the establishment of causal relationships and the comparison of effectiveness between CFT and other antibiotics. In addition, the limited number of deaths required the application of parsimonious multivariate models that might have excluded other potentially relevant variables. A further limitation is the heterogeneity in the clinical application of CFT, including variability in dosage, treatment duration and antibiotic combinations, which reflects real-world clinical practice but hampers direct comparisons across different treatment strategies. Moreover, the use of CFT may be subject to indication bias, as it was frequently prescribed in more severe or salvage therapy cases, which may have influenced the observed outcomes. Study strengths include its sample size (the largest to date in this context) and multi-center real-world design, although further research is needed in wider patient populations. It addresses a gap in evidence regarding the performance of CFT in patients who substantively differ from those in the clinical trial populations.

In conclusion, CFT exhibited a favorable safety profile in the treatment of patients with GP IE. It was generally prescribed in combination with other antibiotics and mainly administered as salvage therapy in highly comorbid and clinically severe patients, many of whom did not undergo surgery when indicated. Around one-third of the patients died from infection, and this mortality was independently associated with the age-adjusted Charlson index and presence of septic shock and MSSA infection. Septic shock and the non-performance of indicated surgery were more frequent in patients with IE caused by MSSA versus other GP microorganisms.

## 4. Materials and Methods

### 4.1. Study Design

A real-life, observational, retrospective and multicenter study was conducted on the effectiveness and safety of CFT in patients with GP-IE treated at three Spanish tertiary-care hospitals in two autonomous regions (Madrid and Andalusia): Hospital Virgen de las Nieves in Granada, Complejo Hospitalario de Jaén, and Hospital Puerta de Hierro in Majadahonda.

The recruitment period extended from the date of the incorporation of CFT in the clinical protocol of the hospitals through May 2024.

The study was approved by the provincial ethics committee of Granada (Ref. 0095-N-22; approved on 27 September 2022) and deemed exempt from the requirement for informed patient consent.

### 4.2. Treatment Description

This descriptive observational study did not require any pharmacological intervention, and antibiotic treatments were always prescribed by attending physicians in accordance with their habitual clinical practice.

### 4.3. Study Population

#### 4.3.1. Inclusion Criteria

Age ≥ 18 years; diagnosis of possible or confirmed GP-IE defined according to modified Duke criteria (2023) [33]; receipt of CFT as first-line or salvage antibiotic treatment for ≥48 h at dosage equivalent to a minimum cumulative dose of 4800 mg in patients with normal renal function, or adjusted for renal clearance in cases of renal impairment.

#### 4.3.2. Exclusion Criteria

Pregnancy; allergy to beta-lactams or any component of the formulation of CFT; IE with no microbiological isolation or caused by microorganisms other than GP.

### 4.4. Variables and Definitions

#### 4.4.1. Variables

Clinical, microbiological, treatment-related, and outcome data were extracted from electronic medical records of the hospitals and processed in accordance with Spanish data protection regulations (Organic Law 3/2018 of December 5 on the Protection of Personal Data and Guarantee of Digital Rights).

Information was gathered on patient age; sex at birth; length of hospital stay; hospital department at CFT initiation; history of heart, respiratory, chronic liver or kidney diseases, neoplasms, HIV infection, parenteral drug use; and the presence of an intracardiac or intravascular device. The age-adjusted Charlson index was calculated to determine the comorbidity burden. The presence of sepsis at baseline was defined by qSOFA ≥ 2 and the presence of septic shock by qSOFA ≥ 2 associated with systolic blood pressure < 90 mmHg.

Data was also collected on IE diagnosis (definite or possible) [18], endocarditis type (native valve, early prosthetic valve, late prosthetic valve, or cardiac device-associated), affected valve, presence of septic embolism, and surgical status (not indicated, indicated and performed, indicated but not performed, or device explantation). The reason for not performing indicated surgery was also recorded (comorbidity burden, poor functional status and hemodynamic instability, or patient refusal).

Microbiological variables included the isolated organism, sample source (blood, valve, or device culture), methicillin-sensitivity of Staphylococcus spp., results of control blood cultures and, when applicable, time to blood culture clearance.

Information was gathered on any previous antibiotic treatment for IE; the reason for prescribing CFT; its administration as first-line or salvage therapy, as empirical or targeted treatment, and as monotherapy or in combination with other antibiotics; number of days of CFT administration; and the total cumulative dose. CFT-related adverse effects were documented, including their type and severity and whether CFT discontinuation was required. Any switch from CFT to another antibiotic during hospitalization was recorded, including the motive and the name of the alternative drug. Information was also collected on the antibiotics used as consolidation therapy and on the total duration of antibiotic treatment against IE.

Data was gathered on the overall mortality and IE-related mortality and on the IE-related mortality before day 15, between days 15 and 28, and between day 29 and 6 months. Information was also recorded on any relapse during the six-month follow-up and on the unfavorable composite outcome.

#### 4.4.2. Definitions

The composite unfavorable outcome was defined by the presence of any of the following events: IE-related mortality, discontinuation of CFT due to adverse effects or infection control failure, or IE relapse during follow-up.

IE-related mortality was defined as mortality directly attributable to IE or its complications during hospitalization or follow-up.

Adverse effects were reported to pharmacovigilance under the yellow card scheme by the attending clinicians as part of their habitual clinical practice. They were classified as follows: Mild, requiring no antidote or treatment, with no prolongation of hospitalization; Moderate, requiring dose adjustment or combination with another drug but not CFT discontinuation, although prolongation of hospitalization or a specific treatment might be necessary; Severe, life-threatening and requiring immediate CFT discontinuation and specific treatment; or Fatal, directly or indirectly contributing to death.

Persistent bacteremia was defined by the persistence of positive blood cultures for the same microorganism after ≥72 h of antibiotic treatment considered effective.

Relapses were defined by the recurrence of IE due to the same microorganism during the six-month follow-up period.

Empirical therapy was defined as initiation of CFT prior to microbiological confirmation, whereas targeted therapy referred to cases in which the microbiological isolate was already known at the time of CFT initiation.

Salvage therapy was defined by the administration of CFT after clinical or microbiological failure, intolerance, or discontinuation of a previous antibiotic treatment.

### 4.5. Sample Size

Given the observational, retrospective, and descriptive study design, sample size estimation was not performed. The study included all consecutive patients who met eligibility criteria during the recruitment period to minimize selection bias and maximize the representativeness of the cohort. Pharmacy departments of the hospitals provided the records of patients who received CFT during the study period.

### 4.6. Statistical Analysis

Data were entered into a pseudo-anonymized database in compliance with Spanish data protection regulations (Organic Law 3/2018, of December 5) and the principles of the Declaration of Helsinki. Statistical analyses were performed using IBM SPSS Statistics for Windows, version 25 (IBM Corp., Armonk, NY, USA). In descriptive analysis, qualitative variables were expressed as absolute and relative frequencies (percentages), and quantitative variables as mean with standard deviation when their distribution was normal and as median with interquartile range (P25–P75) when it was not, as assessed by the Kolmogorov–Smirnov test.

In bivariate analyses of factors associated with IE-related mortality, the chi-square test or Fisher’s exact test was used for qualitative variables, as appropriate, while Student’s t-test was used for quantitative variables with normal distribution and the Mann–Whitney U test for those with non-normal distribution. Variables obtaining a *p*-value ≤ 0.20 or considered clinically relevant were entered into the multivariate analysis.

Because of the limited number of IE-related deaths, a parsimonious binary logistic regression model was constructed to minimize the risk of overfitting. Total CFT dosage, length of hospital stay, and non-performance of indicated surgery were not entered into the final multivariate model to avoid reverse causality bias. Due to the absence of outcome data, three patients (3.9%) were excluded from the outcome analyses; no imputation methods were applied due to the small number of cases.

## Figures and Tables

**Table 1:** Demographic characteristics and comorbidities of patients.

	*N* = 76
Age, mean (years), (±SD)	68.9 (±12.8)
Age-adjusted Charlson comorbidity index, median (IQR)	4 (3–6)
Sex at birth, n (%)	
- Male	50 (65.8)
- Female	26 (34.2)
Cardiovascular risk factors, n (%)	62 (81.6)
- Hypertension	50 (65.8)
- Diabetes mellitus	27 (35.5)
- Dyslipidemia	33 (43.4)
- Obesity	16 (21.1)
- Hyperuricemia, including gout	10 (13.2)
- Obstructive sleep apnea	9 (11.8)
Preexisting cardiovascular disease, n (%)	57 (75.0)
- Moderate or severe valve disease	42 (55.3)
- Atrial fibrillation or flutter	27 (35.5)
- Chronic heart failure	26 (34.2)
- Ischemic heart disease	15 (19.7)
- Cardiac implantable electronic device	6 (7.9)
- Chronic lower limb ischemia	5 (6.6)
Chronic organ diseases, n (%)	
- Chronic obstructive pulmonary disease	10 (13.2)
- Bronchiectasis	3 (3.9)
- Chronic liver disease/cirrhosis	5 (6.6)
- Chronic kidney disease	13 (17.1)
- Hemodialysis	4 (5.3)
- Dementia/cognitive impairment	2 (2.6)
- Stroke	7 (9.2)
Immunosuppression, n (%)	
- Immunosuppressive drug therapy	7 (9.2)
- Active solid malignancy	1 (1.3)
- Active hematologic disease	3 (3.9)
- Allogeneic hematopoietic cell transplantation	2 (2.6)
- Solid organ transplantation	2 (2.6)
- HIV infection (CD < 200/µL), n (%)	2 (2.6)

SD = standard deviation, IQR = interquartile range (P25–P75).

**Table 2:** Characteristics of ceftaroline use and surgical management in infective endocarditis episodes.

	*N* = 76
Ceftaroline exposure	
- Total dose (g), median (IQR)	17.5 (8.6–34.5)
- Days of administration, median (IQR)	14 (6–24)
- Empirical use, n (%)	26 (34.2)
- Targeted use	50 (65.8)
- First-line, n (%)	26 (34.2)
- Second-line or more	50 (65.8)
Previous antibiotic therapy, n (%)	50 (65.8)
Among patients with previous antibiotic therapy (n = 50), n (%)	
- Vancomycin-based regimens	8 (16.0)
- Daptomycin-based regimens	33 (66.0)
- β-lactam-based regimens	25 (50.0)
- Combination therapy (≥2 antibiotics)	25 (50.0)
Days of previous antibiotic therapy, median (IQR)	7 (4–9.25)
Monotherapy vs. combination therapy, n (%)	
- Ceftaroline in monotherapy	4 (5.3)
- Ceftaroline combined with other antibiotics	72 (94.7)
Among patients receiving combination therapy (n = 72):	
- Double Gram-positive antibiotic therapy, n (%)	58 (80.6)
- Ceftaroline + daptomycin	51 (87.9)
- Ceftaroline + cloxacillin	5 (8.6)
- Other dual combinations	2 (3.4)
- Triple Gram-positive antibiotic therapy, n (%)	14 (19.4)
- Ceftaroline + daptomycin + rifampicin	8 (57.1)
- Other triple combinations	6 (42.9)
Reason for switch to ceftaroline, n (%)	50 (65.8)
- Failure of previous antibiotic treatment	32 (42.1)
- Toxicity/adverse events of previous antibiotic treatment	9 (11.8)
- Guided by microbiological results	8 (10.5)
- Not specified	1 (1.3)
Reason for ceftaroline discontinuation or switch during hospitalization, n (%)	28 (36.8)
- Clinical improvement and/or switch to monotherapy	12 (15.8)
- Guided by microbiological results	8 (10.5)
- Toxicity/adverse events of ceftaroline	6 (7.9)
- Lack of microbiological response	1 (1.3)
- Need to broaden antimicrobial spectrum	1 (1.3)
Surgical management, n (%)	
- Valve replacement surgery indicated	49 (64.5)
Among patients with surgical indication (n = 49)	
- Performed	33/49 (67.3)
- Not performed	16/49 (32.7)
Reasons for not replacing valves despite indication (n = 16)	
- Comorbidities and functional status	9 (56.3)
- Hemodynamic instability	5 (31.3)
- Patient refusal	2 (12.5)
Cardiac device removal, n (%)	6 (7.9)
Ceftaroline as complete-course endocarditis therapy, n (%)	9 (11.9)
Switch to oral or long-acting antibiotic as consolidation, n (%)	18 (23.7)
Switch to oral or long-acting antibiotic as chronic treatment, n (%)	4 (5.2)

**Table 3:** Clinical outcomes of infective endocarditis episodes treated with ceftaroline.

	*N* = 76
Length of hospital stay (days), median (IQR)	33 (19.25–48.75)
Mortality, n (%)	
Total mortality	29 (38.2)
Non-infection-related mortality	7 (9.2)
- COVID-19	2 (2.6)
- Ventilator-associated pneumonia	1 (1.3)
- Complication from cardiac surgery (mediastinitis)	1 (1.3)
- Septic shock due to candidemia	1 (1.3)
- In-hospital cardiac arrest (cause not related to endocarditis)	1 (1.3)
- Acute-on-chronic kidney failure (suspected aminoglycoside nephrotoxicity)	1 (1.3)
Infection-related mortality	22 (28.9)
- Death within 14 days	10 (13.2)
- Death within 28 days	8 (10.5)
- Death within 6 months	4 (5.3)
Endocarditis relapse, n (%)	1 (1.3)
Loss to clinical follow-up, n (%)	3 (3.9)
Composite unfavorable outcome (including related-mortality, relapse, and discontinuation for adverse effects or poor control infection), n (%)	31 (40.1)

Causes of non-infection-related mortality were adjudicated by the research team based on clinical judgement and were considered unrelated to infective endocarditis or its antimicrobial treatment.

**Table 4:** Factors associated with infection-related mortality in episodes of infective endocarditis.

	Survivors *N* = 51	Non-Survivors *N* = 22	Bivariate *p* *	Multivariate OR, 95% CI
Age, mean (years), (SD)	67.8 (13.4)	72.6 (11.4)	0.151	
Age-adjusted Charlson comorbidity index, median (IQR)	3.5 (2–5)	6.0 (5–7)	0.0001	1.66 (1.24–2.23)
Sex at birth, n (%)				
- Male	16 (31.4)	8 (36.4)	0.677	
- Female	35 (68.6)	14 (63.6)		
Hospital department at ceftaroline initiation, n (%)				
- Medical department	33 (64.7)	10 (45.5)	0.125	
- Intensive care unit	8 (15.7)	12 (54.5)	0.0001	
- Surgical department	10 (19.6)	0 (0.0)	0.025	
Cardiovascular risk factors, n (%)				
- Hypertension	32 (62.7)	17 (77.3)	0.225	
- Diabetes mellitus	16 (31.4)	11 (50.0)	0.130	
- Obesity	10 (19.6)	6 (27.3)	0.468	
Pre-existing cardiovascular disease, n (%)				
- Chronic heart failure	14 (27.5)	11 (50.0)	0.062	
- Ischemic heart disease	6 (11.8)	9 (40.9)	0.005	
- Moderate to severe valve disease	28 (54.9)	12 (54.5)	0.978	
- Atrial fibrillation or flutter	20 (39.2)	7 (31.8)	0.548	
- Chronic lower limb ischemia	1 (2.0)	4 (18.2)	0.027	
Chronic organ diseases, n (%)				
- Chronic obstructive pulmonary disease	5 (9.8)	5 (22.7)	0.141	
- Chronic liver disease/cirrhosis	2 (3.9)	3 (13.6)	0.157	
- Chronic kidney disease	4 (7.8)	8 (36.4)	0.003	3.86 (0.84–17.80)
- Stroke	3 (5.9)	3 (13.6)	0.357	
Immunosuppression, n (%)				
- Immunosuppressive drug therapy	4 (7.8)	3 (13.6)	0.424	
- Solid organ transplantation	0 (0.0)	2 (9.1)	0.088	
Sepsis (including shock), n (%)	12 (23.5)	11 (50.0)	0.025	
- Septic shock	9 (17.6)	8 (36.4)	0.083	5.42 (1.38–21.36)
Type of endocarditis, n (%)				
- Pacemaker-related endocarditis	5 (9.8)	1 (4.5)	0.661	
- Native	27 (52.9)	13 (59.1)	0.628	
- Early prosthetic	10 (19.6)	1 (4.5)	0.099	
- Late prosthetic	9 (17.6)	7 (31.8)	0.179	
Site of infection, n (%)				
- Aortic valve	16 (31.4)	12 (54.5)	0.062	
- Mitral valve	18 (35.3)	4 (18.2)	0.144	
- Multiple valves involved	9 (17.6)	3 (13.6)	0.671	
Septic emboli, n (%)	29 (56.9)	10 (45.5)	0.370	
Etiology of infective endocarditis, n (%)				
- MRSA	12 (21.6)	4 (18.2)	0.742	
- MSSA	11 (21.6)	10 (45.5)	0.039	3.85 (1.11–13.39)
- CoNS	23 (45.1)	7 (31.8)	0.290	
Persistently positive blood cultures, n (%)	0 (0.0)	4 (18.2)	0.008	
Total dose (g) of ceftaroline, mean (SD)	27.4 (22.2)	16.1 (12.5)	0.007	
Days of administration of ceftaroline, mean (SD)	18.3 (12.8)	10.8 (7.0)	0.002	
Ceftaroline use, n (%)				
- Empirical use	20 (39.2)	4 (18.2)	0.079	
- Targeted use	31 (60.8)	18 (81.8)		
First-line versus salvage therapy, n (%)				
- Ceftaroline as first-line therapy	19 (37.3)	5 (22.7)	0.225	
- Ceftaroline as second-line or more	32 (62.7)	17 (77.3)		
Ceftaroline modality, n (%)				
- Monotherapy	4 (7.8)	0 (0.0)	0.308	
- Combined with other antibiotics	47 (92.2)	22 (100.0)		
Valve replacement surgery indicated, n (%)	28 (54.9)	19 (86.4)	0.010	
- Not performed despite indication	5 (9.8)	9 (40.9)	0.002	
Length of hospital stay, median (IQR)	37.5 (26.25–54.50)	19.50 (12.75–24.50)	0.0001	

CoNS: Coagulase-Negative *Staphylococci*. MRSA: Methicillin-Resistant *Staphylococcus aureus*. MSSA: Methicillin-Sensitive *Staphylococcus aureus*. OR: odds ratio, 95% CI: 95% confidence interval. Analysis restricted to patients with complete follow-up data (n = 73). * *p* < 0.05 significant.

## Data Availability

The data will be made available on reasonable request to the investigators. The authors will provide the journal and reviewers with access to the study database on request.

## Supplementary Materials

The following supporting information can be downloaded at: https://www.mdpi.com/article/10.3390/antibiotics15050466/s1, Table S1: Characteristics of patients with infective endocarditis and hospital location, Table S2: Characteristics of microbiological isolates, Table S3: Adverse drug effects associated with ceftaroline, Table S4: Factors associated with MSSA versus non-MSSA infective endocarditis.

## Abbreviations

The following abbreviations are used in this manuscript:

CFT	Ceftaroline fosamil
GP	Gram-positive
IE	Infective endocarditis
MSSA	Methicillin-sensitive *Staphylococcus aureus*
MRSA	Methicillin-resistant *Staphylococcus aureus*
