# Supplementary material for: Clinical Experience of Ceftaroline Fosamil in Gram-Positive Infective Endocarditis: A Multicenter Real-World Observational Study
Source: Antibiotics (Basel). 2026 May 5;15(5):466. doi: 10.3390/antibiotics15050466 (PMC13203608; doi:10.3390/antibiotics15050466)
Supplement: Supplementary file 1 [file antibiotics-15-00466-s001.zip › Supplementary Table 1. Endocarditis type.pdf]

Supplementary Table 1. Characteristics of patients with infective endocarditis and hospital location

	N = 76
Hospital department at ceftaroline initiation, n (%)	
- Medical department	45 (59.2)
- Intensive care unit	20 (26.3)
- Surgical department	11 (14.5)
Type of endocarditis, n (%)	
- Native	41 (53.9)
- Early prosthetic	11 (14.5)
- Late prosthetic	18 (23.7)
- Pacemaker-related endocarditis	6 (7.9)
Site of infection, n (%)	
- Aortic valve	29 (38.2)
- Mitral valve	23 (30.3)
- Tricuspid valve	2 (2.6)
- Multiple valves involved	12 (15.8)
- Mural endocarditis	1 (1.3)
- Indeterminate	3 (3.9)
Modified Duke criteria, n (%)	
- Definitive	67 (88.2)
- Possible	9 (11.8)
Presence of septic emboli, n (%)	
- No	35 (46.1)
- Yes	41 (53.9)
Presence of sepsis (including septic shock), n (%)	23 (30.3)
- Septic shock	17 (22.4)
