# Supplementary material for: Clinical Experience of Ceftaroline Fosamil in Gram-Positive Infective Endocarditis: A Multicenter Real-World Observational Study
Source: Antibiotics (Basel). 2026 May 5;15(5):466. doi: 10.3390/antibiotics15050466 (PMC13203608; doi:10.3390/antibiotics15050466)
Supplement: Supplementary file 1 [file antibiotics-15-00466-s001.zip › Supplementary Table 2. Microbiology.pdf]

Supplementary Table 2. Characteristics of microbiological isolates

	N = 76
Etiology of infective endocarditis, n (%)	
Gram-positive cocci, n (%)	74 (97.4)
- *Staphylococcus aureus*	37 (50.0)
• MRSA	15 (20.3)
• MSSA	22 (29.7)
- Coagulase-negative S*taphylococcus spp.*	32 (42.1)
• *Staphylococcus epidermidis*	25 (32.9)
• *Other CoNS*	7 (9.2)
- *Enterococcus faecalis*	3 (3.9)
- *Streptococcus pneumoniae*	2 (2.6)
Gram-positive bacilli, n (%)	2 (2.6)
- *Corynebacterium coyleae*	1 (1.3)
- *Cutibacterium acnes*	1 (1.3)
Microbiological isolation sample, n (%)	
- Blood cultures	72 (94.8)
- Valve or cardiac device cultures	4 (5.2)
Microbiological response to therapy, n (%)	
- Control blood cultures performed	69/76 (90.8)
• Persistently positive	4 (5.8)
• Blood culture clearance	65 (94.2)
- Time to blood culture negativity, median (IQR)	4 (2 – 7)

Percentages were calculated for microbiological response variables based on episodes with available follow-up blood cultures (n=69) as denominator. MRSA: methicillin-resistant Staphylococcus aureus, MSSA: methicillin-sensitive Staphylococcus aureus, CoNS: coagulase-negative Staphylococcus
