# Supplementary material for: Clinical Experience of Ceftaroline Fosamil in Gram-Positive Infective Endocarditis: A Multicenter Real-World Observational Study
Source: Antibiotics (Basel). 2026 May 5;15(5):466. doi: 10.3390/antibiotics15050466 (PMC13203608; doi:10.3390/antibiotics15050466)
Supplement: Supplementary file 1 [file antibiotics-15-00466-s001.zip › Supplementary Table 3. Adverse effects.pdf]

Supplementary Table 3. Adverse drug effects associated with ceftaroline

	N = 76
Total adverse effects, n (%)	7 (9.2)
Severity of adverse effects, n (%)	
- Severe	2 (2.6)
- Moderate	4 (5.3)
- Mild	1 (1.3)
Type of adverse effects, n (%)	
- Neutropenia	2 (2.6)
- Gastrointestinal symptoms	2 (2.6)
- Urticaria-like cutaneous rash	1 (1.3)
- Interstitial nephritis	1 (1.3)
- *Clostridioides difficile* infection	1 (1.3)
Interruption of antibiotic treatment due to adverse effects, n (%)	6 (7.9)
