# Supplementary material for: Clinical Experience of Ceftaroline Fosamil in Gram-Positive Infective Endocarditis: A Multicenter Real-World Observational Study
Source: Antibiotics (Basel). 2026 May 5;15(5):466. doi: 10.3390/antibiotics15050466 (PMC13203608; doi:10.3390/antibiotics15050466)
Supplement: Supplementary file 1 [file antibiotics-15-00466-s001.zip › Supplementary Table 4. Bivariate SAMS vs no SAMS.pdf]

Supplementary Table 4. Factors associated with MSSA versus non-MSSA infective endocarditis

	Non-MSSA-IE N = 54	MSSA-IE N = 22	Bivariate p*
Age, mean (years), (SD)	69.3 (11.6)	68.1 (15.4)	0.710
Age-adjusted Charlson comorbidity index, median (IQR)	4 (3 - 6)	4.5 (3 - 6.3)	0.446
Sex at birth, n (%)			
- Male	34 (63.0)	16 (72.7)	0.416
- Female	20 (37.0)	6 (27.3)	
Hospital department at ceftaroline initiation, n (%)			
- Medical department	34 (63.0)	11 (50.0)	0.297
- Intensive care unit	13 (24.1)	7 (31.8)	0.487
- Surgical department	7 (13.0)	4 (18.2)	0.558
Cardiovascular risk factors, n (%)			
- Hypertension	38 (70.4)	12 (54.5)	0.187
- Diabetes mellitus	16 (29.6)	11 (50.0)	0.092
- Obesity	12 (22.2)	4 (18.2)	0.695
Pre-existing cardiovascular disease, n (%)			
- Chronic heart failure	23 (42.6)	3 (13.6)	0.016
- Ischemic heart disease	12 (22.2)	3 (13.6)	0.394
- Moderate to severe valve disease	36 (66.7)	6 (27.3)	0.002
- Atrial fibrillation or flutter	20 (37.0)	7 (31.8)	0.666
- Chronic lower limb ischemia	3 (5.6)	2 (9.1)	0.623
Chronic organ diseases, n (%)			
- Chronic obstructive pulmonary disease	8 (14.8)	2 (9.1)	0.503
- Chronic liver disease / cirrhosis	2 (3.7)	3 (13.6)	0.142
- Chronic kidney disease	8 (14.8)	5 (22.7)	0.406
- Stroke	5 (9.3)	2 (9.1)	1.000
Immunosuppression and oncologic disease, n (%)	5 (9.3)	2 (9.1)	1.000
Sepsis (including shock), n (%)	12 (22.2)	11 (50.0)	0.017
Septic shock	9 (16.7)	8 (36.4)	0.062
Type of endocarditis, n (%)			
- Pacemaker-related endocarditis	4 (7.4)	2 (9.1)	0.805
- Native	26 (48.1)	15 (68.2)	0.112
- Early prosthetic	11 (20.4)	0 (0.0)	0.022
- Late prosthetic	13 (24.1)	5 (22.7)	0.900
Site of infection, n (%)			
- Aortic valve	24 (44.4)	5 (22.7)	0.077
- Mitral valve	15 (27.8)	8 (36.4)	0.460
- Multiple valves involved	9 (16.7)	3 (13.6)	0.742
Septic emboli, n (%)	26 (48.1)	15 (68.2)	0.112
Persistently positive blood cultures, n (%)	2 (3.8)	2 (9.5)	0.574
Total dose (g) of ceftaroline, median (IQR)	24 (9 - 46.8)	12.1 (5.9 - 17.1)	0.016
Days of administration of ceftaroline, median (IQR)	15.0 (7.0 - 28.0)	8.5 (5.0 - 15.5)	0.080
Ceftaroline use, n (%)			
- Empirical use	21 (38.9)	5 (22.7)	0.178
- Targeted use	33 (61.1)	17 (77.3)	
First-line versus salvage therapy, n (%)			
- Ceftaroline as first-line therapy	20 (37.0)	6 (27.3)	0.416
- Ceftaroline as second-line or more	34 (61.8)	16 (72.7)	
Ceftaroline modality, n (%)			

- Monotherapy	4 (7.4)	0 (0.0)	0.317
- Combined with other antibiotics	50 (92.6)	22 (100.0)	
Valve replacement surgery indicated, n (%)	34 (63.0)	15 (68.2)	0.666
- Not performed despite indication	8 (14.8)	8 (36.4)	0.037
Length of hospital stay, median (IQR)	30 (19.0 - 48.0)	33.5 (12.8 - 51.5)	0.722
Total mortality, n (%)	18 (34.6)	11 (52.4)	0.160
- Infection-related mortality	12 (23.1)	10 (47.6)	0.039
Relapse of endocarditis, n (%)	1 (1.9)	0 (0.0)	1.000
